# Editorial: Emerging swine viral pathogens: virus evolution, epidemiology, molecular pathogenesis and disease control strategies

**DOI:** 10.3389/fmicb.2023.1274347

**Published:** 2023-08-25

**Authors:** Karam Chand

**Affiliations:** Division of Virology, ICAR-Indian Veterinary Research Institute, Nainital, Uttarakhand, India

**Keywords:** swine, emerging virus, evolution, vaccine, epidemiology

In past three to four decades emerging viral pathogens of swine have been detected significantly higher than any other species. Among these swine viruses porcine reproductive and respiratory syndrome virus (PRRSV), enteropathogenic porcine epidemic diarrhea virus (PEDV), porcine circovirus type 2 (PCV-2) and influenza A virus (H1N1) are endemic in swine population and continue to challenge researchers, veterinarians and producers. Other viruses, like porcine kobuvirus (PKBV), porcine sapelovirus (PSV), porcine toroviruses (PToV), porcine enteroviruses, porcine bocavirus (PBoV), and porcine Torque teno sus virus (TTSuV) are present mostly in subclinical form in swine population. However, threat persists for these viral pathogens to cause economic impact to the global swine trade. Frequent outbreaks of African swine fever virus (ASFV) and classical swine fever virus (CSFV) have also severely impacted the global swine market. Moreover, novel emerging viruses like Seneca Valley Virus (SVV), porcine circovirus type 3 (PCV-3), atypical porcine pestivirus (APPV), Swine acute diarrhea syndrome coronavirus (SADS-CoV) and influenza D with local or worldwide distribution constitute a recent challenge. Emergence of swine viral pathogen can be explained through factors like, interspecies transmission between aquatic birds and human beings (e.g., influenza A) and change in virulence due to mutation or reassortment (e.g., influenza A H1N1 and PEDV).

This Research Topic consists of four original research article about characterization of PRRSV and Rotavirus group A (RVA), new insights into the design and development of an ASFV subunit vaccine and new strategies to prevent and treat PEDV infection.

Manjate et al. characterized the Rotavirus G3P[8] strain in vaccinated children in Manhiça District, Mozambique. Two Rotavirus G3P[8] strains were selected for whole genome sequencing and characterization because of their emergence and higher frequency in surveillance. The two G3P[8] strains were of Wa-like genotype constellation (G3P[8]-I1-R1-C1-M1-A1-N1-T1-E1-H1) and shared 100% nucleotide (nt) and amino acid (aa) identities in 10 gene segments, except for VP6. Phylogenetic analysis demonstrated that genome segments encoding VP7, VP6, VP1, NSP3, and NSP4 of the two strains clustered most closely with porcine, bovine, and equine strains. The identification of segments exhibiting the closest relationships with animal strains shows significant diversity of rotavirus and suggests the possible occurrence of reassortment events between human and animal strains. Identification of segments exhibiting the closest relationships with animal strains shows significant diversity of rotavirus and may indicate that even with the high efficacy of rotavirus vaccines in reducing severe cases, rotavirus strains continue to evolve, and novel strains still emerge.

Li et al. characterized a novel emerging PRRSV from three different regions of Shandong Province of China. These strains presented a novel deletion pattern (1 + 8 + 1) in the NSP2 region and belonged to a new branch in sublineage 8.7. Based on the phylogenetic analysis of the whole genome, these strains formed a new independent branch in sublineage 8.7, which showed a close relationship with HP-PRRSV and intermediate PRRSV according to nucleotide and amino acid homology but displayed a completely different deletion pattern in NSP2. The results showed that the new-branch PRRSV strains may have the same origin and be similar to HP-PPRSV also evolved from intermediate PRRSV, but are distinct strains that evolved simultaneously with HP-PRRSV. They persist in some parts of China through rapid evolution, recombine with other strains and have the potential to become epidemic strains.

Zhang W. et al. investigated anti-PEDV effects and potential mechanisms of fangchinoline (Fan) as new antiviral measures for PED control. Fan dose-dependently inhibited a PEDV infection at 24 h post-infection. They found that Fan mainly affected the PEDV replication phase but also inhibited PEDV at the attachment and internalization stages of the viral life cycle. Fan blocked the autophagic flux in PEDV-infected cells by regulating the expression of autophagy-related proteins and changing PEDV virus particles.

Zhang H. et al. attempted a novel method for inducing a mucosal immune response against ASFV to prevent ASFV infection through mucosal epithelial cells. They expressed the p30, p54, and p72 proteins encoded by ASFV *in vitro* using the *Lactobacillus lactis* (*L. lactis*) expression system. Following oral immunization of rabbits with recombinant *L. lactis*, serum IgG, intestinal mucosal sIgA, cytokines (IL-4 and INF-γ), and splenocyte viability were higher than in the control group. Oral administration of recombinant *L. lactis* significantly increased rabbits' resistance, including their humoral, cellular, and mucosal immune systems.

In conclusion, this Research Topic provides information about characterization of a novel emerging PRRSV in China and Rotavirus G3P[8] strain in vaccinated children in Manhiça District, Mozambique. A research study showed the anti-PEDV effects and potential mechanisms of fangchinoline (Fan) as new antiviral measures for PED control. Finally, a study shows an oral immunization of rabbits with ASFV recombinant *L. Lactis* induced immunity responses.

## Author contributions

KC: Writing—original draft.

